# Species Distribution Models and Ecological Suitability Analysis for Potential Tick Vectors of Lyme Disease in Mexico

**DOI:** 10.1155/2012/959101

**Published:** 2012-02-14

**Authors:** Patricia Illoldi-Rangel, Chissa-Louise Rivaldi, Blake Sissel, Rebecca Trout Fryxell, Guadalupe Gordillo-Pérez, Angel Rodríguez-Moreno, Phillip Williamson, Griselda Montiel-Parra, Víctor Sánchez-Cordero, Sahotra Sarkar

**Affiliations:** ^1^Section of Integrative Biology, University of Texas at Austin, Austin, TX 78712, USA; ^2^Department of Pathology, Microbiology and Immunology, School of Veterinary Medicine, University of California Davis, Davis, CA 95616, USA; ^3^Centro Médico Nacional SXXI, Unidad de Investigación Médica de Enfermedades Infecciosas y Parasitarias, IMSS, Avenida Cuauhtémoc 330, Colonia Doctores 06725 México, DF, Mexico; ^4^Laboratorio de Sistemas de Información Geográfica, Departamento de Zoología, Instituto de Biología, UNAM, Circuito Exterior, Apartado Postal 70-153, Coyoacán, 04510 México, DF, Mexico; ^5^Department of Forensic and Investigative Genetics, University of North Texas Health Science Center, 3500 Camp Bowie Boulevard, Fort Worth, TX 76107, USA; ^6^Colección Nacional de Ácaros, Departamento de Zoología, Instituto de Biología, UNAM, Circuito Exterior, Apartado Postal 70-153, Coyoacán, 04510 México, DF, Mexico

## Abstract

Species distribution models were constructed for ten *Ixodes* species and *Amblyomma cajennense* for a region including Mexico and Texas. The model was based on a maximum entropy algorithm that used environmental layers to predict the relative probability of presence for each taxon. For Mexico, species geographic ranges were predicted by restricting the models to cells which have a higher probability than the lowest probability of the cells in which a presence record was located. There was spatial nonconcordance between the distributions of *Amblyomma cajennense* and the *Ixodes* group with the former restricted to lowlands and mainly the eastern coast of Mexico and the latter to montane regions with lower temperature. The risk of Lyme disease is, therefore, mainly present in the highlands where some *Ixodes* species are known vectors; if *Amblyomma cajennense* turns out to be a competent vector, the area of risk also extends to the lowlands and the east coast.

## 1. Introduction

Lyme disease, the most frequently reported tick-borne infectious disease in the United States and Europe [1, 2], is increasingly being reported from Mexico [3, 4], where disease cases are more prevalent during warm-weather months when ticks are active. The etiologic agent, *Borrelia burgdorferi*, enters the skin at the site of the tick bite; after incubating for 3–30 days, the bacteria migrate through the skin and may spread to lymph nodes or disseminate through the bloodstream to other parts of the body. While *B. burgdorferi* infection might be endemic in Mexico [3, 4] it is relatively rare in the southern USA making the question of its biogeography a matter of interest.

 Additionally, in Mexico, the epidemiology and biogeography of Lyme disease are not well understood [5]. Several tick species have recently been identified as containing *B. burgdorferi *using a DNA polymerase chain reaction and, therefore, may be considered as candidates that may be involved in the enzootic transmission cycle in both Mexico and South America. These include tick species from the genus *Ixodes *[3, 4] as well as *Amblyomma cajennense* [5, David Beck, personal communication]. While detection of *B. burgdorferi* DNA by polymerase chain reaction is not indicative of vector competence, the presence of *B. burgdorferi* in the molecular surveys does indicate a benefit from modeling the distribution of *A. cajennense* since it has been shown to feed on reservoirs for *B. burgdorferi *in Mexico. Additionally, the South American* A. cajennense* has been shown to be a competent vector for *Rickettsia rickettsii *[6], the causative agent of Rocky Mountain spotted fever, and has been shown to carry additional *Rickettsia* species which belong to the spotted fever group [7].

*Ixodes *ticks are hematophagous parasites during all active life stages. They have great importance from economic, veterinary, and human health vantage perspectives because of their capacity to transmit a variety of diseases to humans and animals [8]. These species are parasites of birds or mammals. In Mexico, 26 *Ixodes *species have been identified; these were collected from 20 of Mexico 32 states [9]. The distribution of *A. cajennense *extends from the southern regions of the United States (Texas) to the Caribbean Islands, and across Central and South America to northern Argentina, excluding the mountain regions [10, 11]. As a consequence, if *A. cajennense* was to contribute to maintenance of *B. burgdorferi *in the zoonotic cycle in any way or be a competent vector for a variety of spotted fevers in Mexico, the health impact could be significant. Thus far, *A. cajennense* has not been found north of latitude 27°N or south of latitude 29°S and its geographic range may be limited by temperature [10]. Low temperatures in mountainous areas such as the Mexican Sierra Madre and the Andes may be an obstacle for its establishment. With this restriction, the species is known to survive in regions with very different ecological conditions, spanning from arid grasslands to tropical forests [10].

 The purpose of this paper is to explore the biogeography of *Ixodes* ticks and *A. cajennense* in Mexico and the suitability of different ecoregions and habitat types to their potential establishment using species distribution models (SDMs). This technique has been systematically developed to explore vector-borne zoonotic disease ecology and biogeography during the last 15 years [12, 13], and several studies have applied it to Mexico and nearby regions [14–16]. The goal was to determine the ecological variables that best predict georeferenced distributional data of a species collected through fieldwork, from museum collections, and so forth. These predictive variables are interpreted as identifying the potential geographical distribution of a species [17] and are sometimes also interpreted as identifying its fundamental niche [14, 18–20]. When biogeographic, behavioral, and other limitations to dispersal are taken into account, the potential distribution is refined to a predicted (realized) distribution.

 For species that are relevant to the transmission of a disease, the relative suitability of different regions within the predicted distribution, as measured on a continuous scale, establishes the relative spatial ecological risk [13, 16, 17]. For vector-borne zoonotic diseases, a composite measure for this risk must include the SDMs of all relevant vector and reservoir species. This risk can then be combined with other measures of risk, including socioeconomic factors and disease case prevalence. A variety of techniques have been developed to carry out such increasingly sophisticated disease risk analyses [17].

 Because of a lack of data on other factors, this study is restricted to SDMs for potential tick vectors of Lyme disease. The aim was to analyze the predicted biogeography and habitat suitability for the *Ixodes* species, treated jointly, and *A. cajennense*. *Ixodes *species seem to be the most likely candidates for the transmission of Lyme disease in Mexico, and *A. cajennense *has been shown to be a competent vector for multiple tick-borne rickettsioses. Besides establishing the relative risk of the transmission of these diseases from these taxa, these SDMs will also permit prediction of the distributions of potentially epidemiologically relevant vector and reservoir distributions. This will allow the identification of the most likely candidates to transmit *B. burgdorferi* infections so that future studies can be guided by a better theoretical understanding of the underlying ecology of Lyme disease in Mexico.

 A wide variety of techniques exist for SDM construction [21]. If presence-only (rather than presence-absence) data are all that are available, as is typically the case (including this study), machine-learning algorithms provide the most reliable results [21, 22]. These use georeferenced data on species occurrence points and environmental layers as input variables; as output they either provide binary predictions of presence or absence or a continuous measure than can be interpreted as relative habitat suitability. For risk analysis the latter is preferable. For this study, we chose a maximum entropy algorithm implemented in the MaxEnt software package [23–25] because, besides providing continuous output, its performance has been established as being as good or better than available alternatives [21, 22]. This choice has also become standard in constructing SDMs for systematic conservation planning [26–28].

## 2. Materials and Methods

### 2.1. Data

#### 2.1.1. Tick Occurrence Data

Tick occurrence data were compiled from various sources including new field collections and information from prior publications. The field data were obtained from the University of North Texas Health Science Center, The University of Texas at Austin, the Texas Department of State Health Services (TX DSHS) and the Instituto de Biología, UNAM, Mexico. Specimens were identified by morphologic examination and by PCR amplification of 12S rDNA followed by sequence determination of the amplification products using the method of Williamson et al. [29]. All points were georeferenced using the MaNIS protocol (http://manisnet.org/GeorefGuide.html, last accessed 19 June 2011). Additional data came from Dergousoff et al. [30].

 SDMs were constructed for an area including Mexico and Texas, both of which had sparse occurrence records; there were insufficient data to construct reliable models for Mexico or Texas alone.  Table 1 lists all the data that were available for all species in Mexico and Texas and is restricted to those used in this analysis, along with the number of points that satisfied the error constraint (see Section 2.2) and the number of such points in independent cells. All data have been submitted to the Disease Vectors Database [31]. Given that the area of epidemiological interest for this paper was Mexico, the model results that were subjected to further analysis and are presented here are for Mexico.

#### 2.1.2. Environmental Layers

The environmental layers used are listed in Table 2. These include four topographical variables (elevation, slope, aspect, and compound topographical index) and 19 bioclimatic variables. The latter were obtained from the WorldClim database [32] (http://www.worldclim.org/; last accessed 28 February 2010). Elevation data were obtained from the United States Geological Survey Hydro-1K DEM data set (http://eros.usgs.gov/#/Find_Data/Products_and_Data_Available/gtopo30/hydro; last accessed 28 February 2010). Slope, aspect, and the compound topographical index were derived from the DEM using the Spatial Analyst extension of ArcMap 9.3.

### 2.2. Species Distribution Models

The study area of Mexico and Texas was divided into 3 429 052 cells at a resolution of 30 arcseconds. The average cell area was 0.77 km^2^. Data were retained for this analysis only if the estimated error was less than 1 arcminute. Data prior to 1990 was excluded from the present analysis. Table 2 shows the number of data that were retained. A conservative threshold of independent data points (i.e., those falling in different cells at the resolution of this analysis) was used for model construction, namely, at least 10 independent cells [17]. 

 SDMs were constructed separately for *A. cajennense*, but for together 10 *Ixodes* species (*I. boliviensis, I. conepati, I. cookie, I. eadsi, I. kingi, I. marxi, I. scapularis, I. sculptus, *and* I. texanus*) for three reasons: (i) though from this group only* I. scapularis* has so far been implicated as a vector for Lyme disease, other *Ixodes* species (e.g., *I. pacificus* and those of the *I. ricinus* complex) are also confirmed vectors. Consequently, it remains possible that these others may be competent vectors. (ii) The provenance of data points suggested that several of these species often cooccur (e.g., *I. scapularis* and *I. sculptus* in Texas). Given the sparse data points available, this meant that the geographical range of these potential vectors may be significantly underestimated if the SDMs were constructed separately for each species. (iii) Treating the data points together allowed much more reliable SDM construction because of the higher number of data points available for input.

 Following a standard protocol [17], MaxEnt (Ver. 3.3.4) was run with the threshold and hinge features and without duplicates so that there was at most one sample per pixel; linear, quadratic, and product features were used. The convergence threshold was set to a conservative 1.0 × 10^−5^. For the AUC, that is, the area under the receiver-operating characteristic (ROC) curve, averages over 100 replicate models were computed. For each model the test : training ratio was set to 40:60 following Phillips and Dudìk [25] which means that models were constructed using 60% of the data and tested with the remaining 40%. An acceptability threshold of 0.90 was used for both the test and training AUCs, well above the standard 0.60 used in the literature.

 Obtaining predicted ranges for the sake of comparisons required the conversion of SDM outputs, which were relative probabilities (specifying habitat suitability) into binary predictions of presence or absence. This was done using a threshold of 0.10 for *A. cajennense* and 0.12 for the *Ixodes* group which corresponded to the lowest probability predicted by the SDMs for an occurrence point used in model construction. The threshold was used after normalization of the MaxEnt output in Mexico so that the highest predicted value for occurrence in each model was 1 for at least one cell in the landscape.

## 3. Results and Discussion

### 3.1. Species Distribution Models

**Figure 1:**
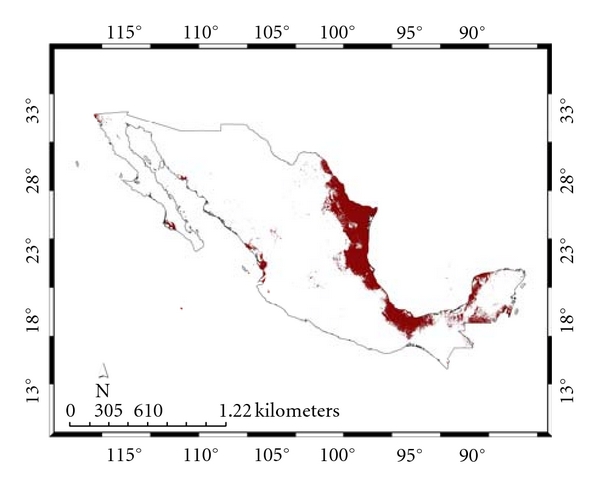
Species distribution model for *Amblyomma cajennense*.

**Figure 2:**
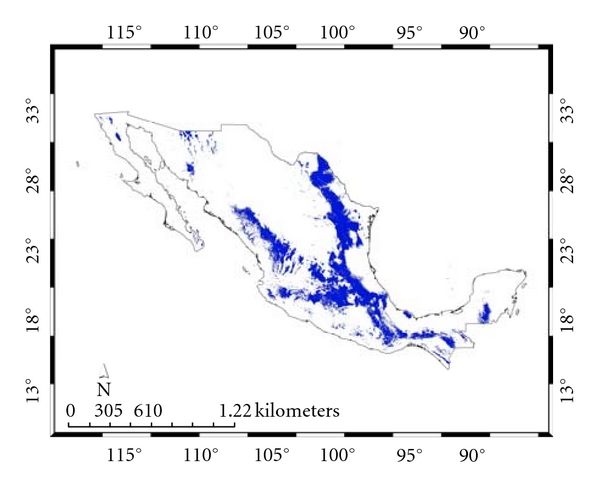
Species distribution model for the *Ixodes* group.

**Figure 3:**
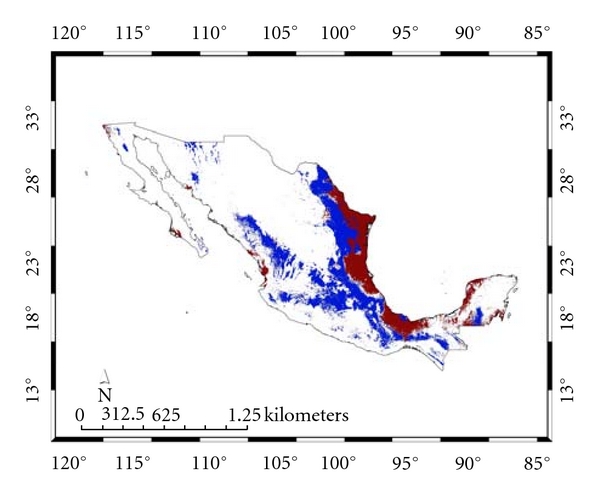
Complementarity of models for *Amblyomma cajennense *and the *Ixodes* group.

Figure 1 presents the species distribution model for* A. cajennense* Figure 2 and that for the present *Ixodes *group. For the 100 replicate models, for* A. cajennense*, the average test AUC was 0.91, the training was 0.99; for the *Ixodes *group, the corresponding numbers were 0.93 and 0.98.  Figure 3 presents both distributions together showing their almost complete nonconcordance, which we will refer to as their “complementarity.”

**Figure 4:**
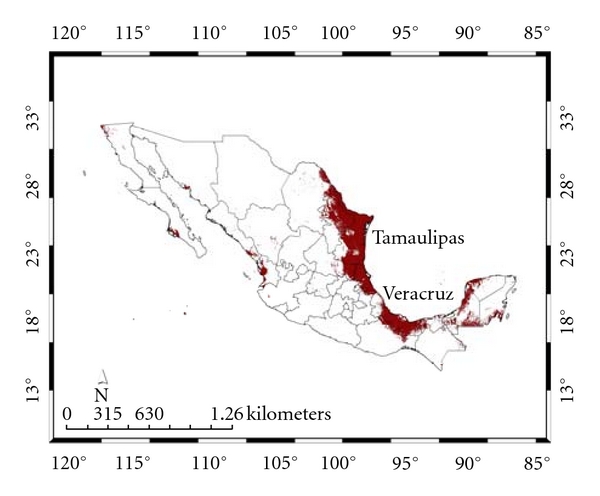
Species distribution model and Mexican states for *Amblyomma cajennense*. The principal states are shown (see text).

**Figure 5:**
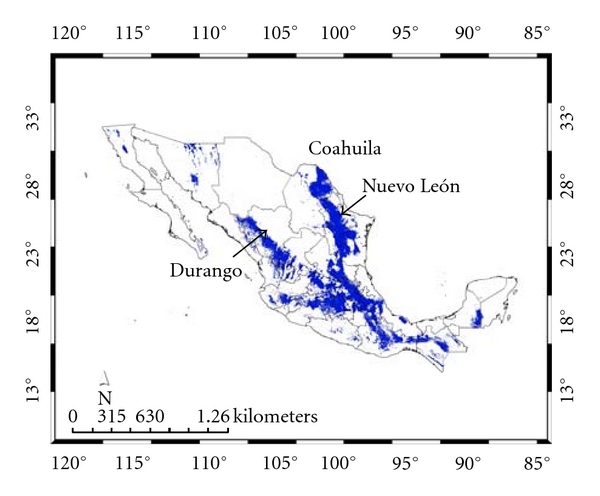
Species distribution model and Mexican states for the *Ixodes* group. The principal states are shown (see text).

Table 3 presents the areas occupied by the predicted distributions for the states of Mexico (see, also, Figures 4 and 5). The *Ixodes *group is predicted to be present in all states, while *A. cajennense* is predicted for all of them except Aguascalientes, Distrito Federal (Mexico City), Morelos, and Tlaxcala, all of which are located in central Mexico. The main distribution predicted for *A. cajennense* is in Veracruz (21.8%) and Tamaulipas (27.8%) (Figure 4), both in the northeast coast of Mexico and both having lowlands and warm temperatures [32]. In contrast, the *Ixodes* group is predicted mainly in Durango (8.7%), Coahuila (9.6%), Nuevo León (9.9%) (Figure 5), and all of the northern states characterized by the presence of high altitudes and temperate vegetation (see below).

### 3.2. Ecological Suitability

Table 4 presents the altitudinal dependence of the two SDMs. Although the complete predicted *A. cajennense *range is between 0 and 2800 m, most of it (95%) occurs between 200 and 1000 m. This result agrees with Solís [33] who found this species only in areas with altitudes below 1000 m even though, geographically, the species is widely distributed in the warmer parts of Latin America and the Caribbean [33]. However, in Guatemala, an ecological and epidemiological study of ticks [34] recorded that the presence of *A. cajennense *occurs up to 1400 m in areas with a marked rainy season (six months of rain and six months for dry season) [35]. The SDMs predict an expanded altitudinal range while confirming that the best habitat is between 200 and 1000 m.

 For the *Ixodes *group (Table 4), the complete altitudinal range goes from 200 m to over 5000 m though most of it (98%) is restricted to below 3600 m. The altitudinal range of the *Ixodes* group thus also complements that of *A. cajennense*, partly accounting for the geographical complementarity noted earlier.

**Figure 6:**
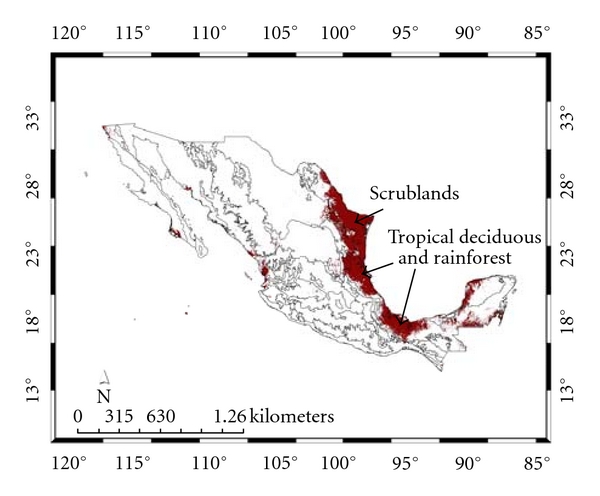
Species distribution model and ecoregions for *Amblyomma cajennense*. The principal ecoregions are shown (see text).

**Figure 7:**
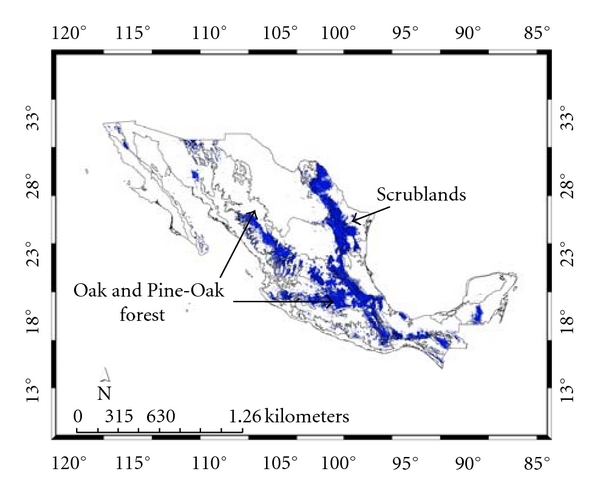
Species distribution model and ecoregions for the *Ixodes* group. The principal ecoregions are shown (see text).

Table 5 shows the ecoregional distribution of the two SDMs (see, also, Figures 6 and 7). Although both SDMs share ecoregions, *A. cajennense* presence was primarily predicted for ecoregions such as mangroves and marshes along the coast of Mexico at low altitudes (Figure 6). In Mexico and the United States, this species is found in areas where the mean temperature is around 13°–16°C and the NDVI is high [36]. Relatively low mean temperatures and differences in the seasonal patterns of rainfall may limit this species colonization of areas to the north of its current distribution. Low temperatures are likely keeping the species out of elevated areas, such as the Sierra Madre in Mexico. The southern distribution of *A. cajennense *appears to be mainly restricted by relatively low temperatures and not by low humidity [36].

**Figure 8:**
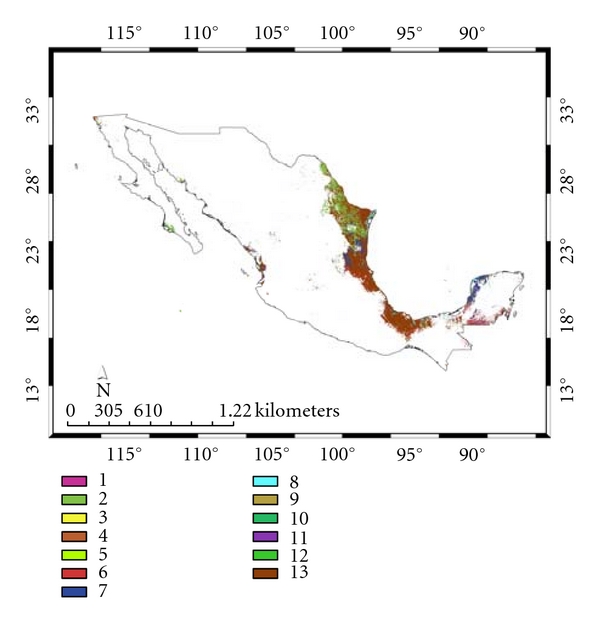
Species distribution model and vegetation types for *Amblyomma cajennense*: (1) grassland; (2) scrubland; (3) pine forest; (4) oak forest; (5) pine-oak forest; (6) tropical rainforest; (7) tropical deciduous forest; (8) aquatic inland vegetation; (9) cloud forest; (10) mangle; (11) palms/palm plantations; (12) savanna; (13) other vegetation types/not known.

**Figure 9:**
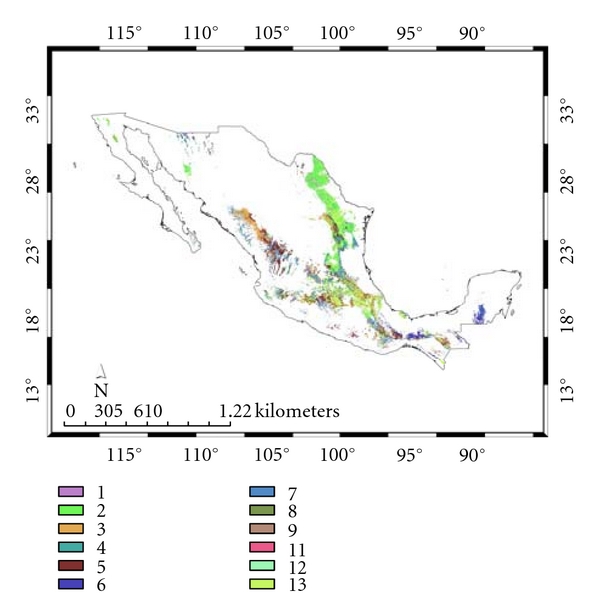
Species distribution model and vegetation types for the *Ixodes* group: (1) grassland; (2) scrubland; (3) pine forest; (4) oak forest; (5) pine-oak forest; (6) tropical rainforest; (7) tropical deciduous forest; (8) aquatic inland vegetation; (9) cloud forest; (10) mangle; (11) palms/palm plantations; (12) savanna; (13) other vegetation types/not known.

Table 6 shows the different vegetation types associated with both models (see, also, Figures 8 and 9). Although both SDMs share scrubland as the main vegetation type, 18.7 and 20.0%, respectively, for *A. cajennense *and the *Ixodes* group, the former is mainly associated with tropical deciduous and rainforest (17.4%), while the latter is associated with oak and pine-oak forest (23.3%). These predictions agree with Álvarez et al. [35] who collected *A*. *cajennense *in tropical wet forests and its transitions. It is likely that suitable *A*. *cajennense *habitat consists of warmer areas with moderate precipitation.

Moreover, suitable *A. cajennense* habitat is predicted to be restricted to areas with more dense or mixed vegetation and tall grass [37]. A study of horse farms showed that pastures were most likely to be infested with *A. cajennense* when the pasture had mixed vegetation (grasses and shrubs) and was cut less than once per year [38]. In Argentina, *A. cajennense* was more abundant in forested areas than open areas [39]. In contrast, species from the *Ixodes* group are typically collected in heavily forested or dense brushy areas.

## 4. Conclusion

Species distribution models are potentially a powerful tool for assessing risk from vector-borne diseases [12, 17]. Even in systems as poorly understood as the one examined here, patterns of concordance in geographic or ecologic space can provide testable hypotheses for host, vector, and reservoir interactions besides their associations with habitat type, vegetation, or ecoregion. Such distributional hypotheses can form the basis for field studies, including analyses of specific parameters of species ecologic niches [40, 41], prediction of species distributions across scenarios of climate change [14, 42, 43], prediction of species invasions [9, 17, 44, 45], assessment of patterns of evolutionary change in ecologic parameters [46], and spatial/epidemiologic stratification of disease endemic areas.

 Little is known about Lyme disease and its transmission cycle in Mexico. Assuming that the *Ixodes* group contains the vectors responsible for transmission, the results presented here identify the geographical regions and ecological characteristics of the regions with the highest potential for transmission: high-altitude low-temperature areas. The SDM also suggests why Lyme disease is relatively rare in the southern United States: the high temperatures of these areas make them relatively less suitable for potential *Ixodes* vectors.

 Should *A. cajennense* affect the enzootic transmission cycle and assist with maintenance of *B. burgdorferi* in reservoir species, the area of high risk extends into the eastern lowlands of Mexico where the SDM for this species complements that of the *Ixodes* group. This result suggests that it is important to test *A. cajennense* for vector competence using appropriate laboratory methods.

 Field efforts are currently under way to collect specimens of potential mammal reservoirs of *B. burgdorferi *and *R. rickettsii*. SDMs of these species will permit analysis of spatial correlations between them and the vector SDMs which will permit the formulation of testable hypotheses about the Lyme disease cycle in Mexico.

## Figures and Tables

**Table 1:** Total number of records and final number of records used to generate the analysis.

Tick species	Mexico			Texas		
	Total number	Number with adequate precision	Independent cells	Total number	Number with adequate precision	Independent cells
*Amblyomma cajennense*	10	10	9	269	269	69
*Ixodes boliviensis*	10	1	1	0	0	0
*Ixodes conepati*	2	2	2	0	0	0
*Ixodes cookei*	3	3	3	0	0	0
*Ixodes eadsi *	5	4	4	0	0	0
*Ixodes kingi*	1	1	1	0	0	0
*Ixodes marxi*	1	1	1	0	0	0
*Ixodes scapularis*	5	4	3	56	56	51
*Ixodes sculptus*	0	0	0	1	1	1
*Ixodes texanus*	2	1	1	0	0	0

**Table 2:** Environmental parameters for species distribution models.

Parameters
Annual mean temperature
Mean diurnal range
Isothermality
Temperature seasonality
Maximum temperature of warmest month
Minimum temperature of coldest month
Temperature annual range
Mean temperature of the wettest quarter
Mean temperature of the driest quarter
Mean temperature of the warmest quarter
Mean temperature of the coldest quarter annual precipitation
Precipitation of wettest month
Precipitation of driest month
Precipitation seasonality
Precipitation of wettest quarter
Precipitation of driest quarter
Precipitation of warmest quarter
Precipitation of coldest quarter
Elevation
Slope
Aspect
Compound topographic index

**Table 3:** Size (area) of predicted range of *Amblyomma cajennense* and the *Ixodes* group.

State	*Amblyomma cajennense*		*Ixodes*	
	No. cells	Area (km^2^)	No. cells	Area (km^2^)
Aguascalientes	0	0	1226	944.02
Baja California	1995	1536.15	3081	2372.37
Baja California Sur	2467	1899.59	1230	947.1
Campeche	21243	16357.11	7602	5853.54
Chiapas	2238	1723.26	17743	13662.11
Chihuahua	70	53.9	2380	1832.6
Coahuila	11578	8915.06	41080	31631.6
Colima	114	87.78	152	117.04
Distrito Federal	0	0	785	604.45
Durango	986	759.22	37042	28522.34
Estado de Mexico	8	6.16	18010	13867.7
Guanajuato	654	503.58	20401	15708.77
Guerrero	37	28.49	4747	3655.19
Hidalgo	997	767.69	19004	14633.08
Jalisco	542	417.34	20600	15862
Michoacán	39	30.03	22676	17460.52
Morelos	0	0	490	377.3
Nayarit	6087	4686.99	3415	2629.55
Nuevo León	43863	33774.51	42073	32396.21
Oaxaca	11002	8471.54	32519	25039.63
Puebla	2594	1997.38	18786	14465.22
Querétaro	204	157.08	10271	7908.67
Quintana Roo	6338	4880.26	1307	1006.39
San Luis Potosí	13836	10653.72	17477	13457.29
Sinaloa	2683	2065.91	3716	2861.32
Sonora	1409	1084.93	12211	9402.47
Tabasco	7571	5829.67	147	113.19
Tamaulipas	80607	62067.39	29230	22507.1
Tlaxcala	0	0	4571	3519.67
Veracruz	63260	48710.2	14420	11103.4
Yucatán	7966	6133.82	99	76.23
Zacatecas	4	3.08	17027	13110.79

**Table 4:** Altitudinal intervals and predicted ranges of *Amblyomma cajennense* and the *Ixodes* group.

Interval	*Amblyomma cajennense*		*Ixodes*	
	No. cells	Area (km^2^)	No. cells	Area (km^2^)
1–200	148	113.96	0	0
201–400	13833	10651.41	5382	4144.14
401–600	6189	4765.53	16702	12860.54
601–800	3296	2537.92	16647	12818.19
801–1000	1351	1040.27	13644	10505.88
1001–1200	476	366.52	12412	9557.24
1201–1400	145	111.65	13443	10351.11
1401–1600	106	81.62	15187	11693.99
1601–1800	86	66.22	17187	13233.99
1801–2000	131	100.87	17490	13467.3
2001–2200	92	70.84	22017	16953.09
2201–2400	38	29.26	26877	20695.29
2401–2600	65	50.05	26918	20726.86
2601–2800	56	43.12	19741	15200.57
2801–3000	8	6.16	8194	6309.38
3001–3200	0	0	3201	2464.77
3201–3400	0	0	1449	1115.73
3401–3600	0	0	761	585.97
3601–3800	0	0	450	346.5
3801–4000	0	0	234	180.18
4001–4200	0	0	108	83.16
4201–4400	0	0	59	45.43
4401–4600	0	0	18	13.86
4601–4800	0	0	8	6.16
4801–5000	0	0	3	2.31
>5000	0	0	1	0.77

**Table 5:** Ecoregion occupancy by *Amblyomma cajennense* and the *Ixodes* group.

Ecoregion	*Amblyomma cajennense*		*Ixodes*	
	No. cells	Area (km^2^)	No. cells	Area (km^2^)
Pine and oak forest	3112	2396.24	196510	151312.7
Cloud forest	179	137.83	8913	6863.01
Chaparral	1681	1294.37	777	598.29
Mangrove	10432	8032.64	0	0
Tamaulipan scrub thorn forest	84540	65095.8	46848	36072.96
Submontane scrubland	22664	17451.28	24305	18714.85
Xeric scrubland	9409	7244.93	74934	57699.18
Marshes of Centla	1135	873.95	0	0
Tropical rainforest	110791	85309.07	31043	23903.11
Tropical deciduous forest	46377	35710.29	42190	32486.3
Tropical dry forest	799	615.23	0	0

**Table 6:** Vegetation types for *Amblyomma cajennense* and the *Ixodes* group.

Vegetation type	*Amblyomma cajennense*		*Ixodes*	
	No. cells	Area (km^2^)	No. cells	Area (km^2^)
Grassland	925	712.25	6602	5083.54
Scrubland	9560	7361.2	14920	11488.4
Pine forest	72	55.44	6478	4988.06
Oak forest	392	301.84	7602	5853.54
Pine-oak forest	76	58.52	9654	7433.58
Tropical rainforest	4116	3169.32	2622	2018.94
Tropical deciduous forest	4815	3707.55	6541	5036.57
Aquatic inland vegetation	1330	1024.1	14	10.78
Cloud forest	14	10.78	1501	1155.77
Mangle	473	364.21	0	0
Palms/palm plantations	30	23.1	18	13.86
Savanna	195	150.15	48	36.96
Other vegetation types/not known	28921	22269.17	17818	13719.86
